# Overexpression of *CENPL* mRNA potentially regulated by miR-340-3p predicts the prognosis of pancreatic cancer patients

**DOI:** 10.1186/s12885-022-10450-5

**Published:** 2022-12-26

**Authors:** Zhongyuan Cui, Ling Du, Jielong Wang, Zhongzhuan Li, Jiehong Xu, Shiyu Ou, Dongliang Li, Shasha Li, Hanfang Hu, Gang Chen, Zhixian Wu

**Affiliations:** 1 Department of Hepatobiliary Disease, 900th Hospital of the Joint Logistics Support Force (Dongfang Hospital), Xiamen University, Fuzhou, 350025 Fujian China; 2 Department of Gastroenterology, the Fourth Affiliated Hospital (Liuzhou Workers’ Hospital), Guangxi Medical University, Liuzhou, 545000 Guangxi China; 3 Department of Hepatobiliary Disease, 900th Hospital of the Joint Logistics Support Force, Fujian Medical University, Fuzhou, 350025 Fujian China

**Keywords:** PAAD, *CENPL*, Biomarkers, Prognosis, miR-340-3p

## Abstract

**Background:**

In our previous study it was found that *CENPL* was overexpressed in hepatocellular carcinoma and significantly predicted patient's prognosis. However, the expression and prognostic value of *CENPL* in other gastrointestinal tumors remain unknown. Therefore, we investigated the expression and prognostic value of *CENPL* in esophageal carcinoma (ESCA), stomach adenocarcinoma (STAD), pancreatic adenocarcinoma (PAAD), colon adenocarcinoma (COAD) and rectum adenocarcinoma (READ).

**Methods:**

In this study, Oncomine, GEPIA, OncoLnc, TIMER, cBioPortal, miRWalk and ENCORI databases were used to analyze the level of *CENPL* mRNA, prognostic value and potential regulatory mechanism of *CENPL* mRNA in tumors. The *CENPL* expression and clinicopathological data regarding PAAD were from the UCSC Xena database and univariate and multivariate Cox regression analyses were performed using R (Version 3.6.3). Immunohistochemical staining was used to verify the expression of CENPL protein in clinical specimens. Cytoscape (Version: 3.7.2) was used to visualize microRNA (miRNA) that potentially regulates *CENPL*.

**Results:**

Gene differential expression analysis showed that *CENPL* mRNA was significantly overexpressed in ESCA, STAD, PAAD, COAD and READ (*p* < 0.01). The overexpression of *CENPL* mRNA was significantly correlated with the poor prognosis of PAAD patients (*p* < 0.05). However, there was no significant correlation between the level of *CENPL* mRNA and the prognosis of ESCA, STAD, COAD and READ patients (*p* > 0.05). Univariate and multivariate Cox regression analyses suggested that *CENPL* was a prognostic risk factor for PAAD. The mutation rate of *CENPL* in PAAD was 2.2% (17/850). There was no significant correlation between the *CENPL* expression and the infiltration levels of immune cells in PAAD (|Cor|< 0.5). Immunohistochemical staining showed that CENPL was overexpressed in 42% (11/26) of PAAD specimens, which was significantly higher compared with that in the normal tissues. The expression of miR-340-3p and miR-484 in PAAD were significantly lower than in the normal tissues (*p* < 0.05) and PAAD patients with lower expression of miR-340-3p had poorer prognosis (*p* < 0.05).

**Conclusion:**

CENPL potentially regulated by miR-340-3p, is overexpressed in PAAD and predicts patient’s prognosis, suggestive of a diagnostic and prognostic value in PAAD patients.

**Supplementary Information:**

The online version contains supplementary material available at 10.1186/s12885-022-10450-5.

## Introduction

Gastrointestinal (GI) tumors including esophageal carcinoma (ESCA), stomach adenocarcinoma (STAD), pancreatic adenocarcinoma (PAAD), colon adenocarcinoma (COAD), rectum adenocarcinoma (READ) and liver hepatocellular carcinoma (LIHC) are the major malignancies globally with significant mortality and morbidity [1–6]. Because the treatment for patients with, especially, advanced GI tumors are limited and the patient prognosis is poor [6–9], it is necessary to explore novel diagnostic and therapeutic markers for GI tumors.

Centromere protein L (CENPL) is a member of the centromere protein family. It is assembled with other centromere proteins to form the constitutive centromere associated network (CCAN), also known as inner kinetochore [10–12]. CENP-L-N complex plays an important role in centromere specificity and kinetochore stability [12]. The depletion phenotype of CENP-L is crucial to the stability of kinetochore microtubules, for a homogenous poleward microtubule flux rate and for the kinetochore pushing force [13]. These studies suggest that CENPL is indispensable during normal cell division. However, the understanding of CENPL in cancer is still limited. Yin et al. found that CENPL might be a diagnostic and prognostic marker for breast cancer [14]. In our previous study it was found that CENPL was overexpressed in hepatocellular carcinoma and predicted the prognosis of patients, and the overexpression of CENPL was positively correlated with the abundance of various tumor infiltrating lymphocytes [15]. Whether *CENPL* plays a role in the development and progression of other GI tumors remains unknown, and more studies are needed.

In this study, we investigated the expression and prognostic value of CENPL in GI tumors, including ESCA, STAD, PAAD, COAD and READ, by using bioinformatic methods and immunohistochemical staining.

## Methods

### Analysis of *CENPL* gene expression

The level of *CENPL* mRNA in esophageal, gastric, colon and pancreatic tumors and adjacent normal tissues was analyzed with Oncomine (https://www.oncomine.org/) [16] and GEPIA: Gene Expression Profiling Interactive Analysis (http: //gepia.cancer-pku.cn) [17] databases. The default settings were used in all analyses, and Fold Change (FC) of ≥ 2 and a *p* value of < 0.05 were considered statistically significant.

### Gene expression and clinical characteristics

OncoLnc (http://www.oncolnc.org/) [18] and GEPIA database were used to analyze the prognosis of patients with ESCA, STAD, PAAD, COAD and READ. For each cancer, the patients were divided into high- and low-level groups according to the gene expression level, and the cutoff was 50%. UALCAN (http://ualcan.path.uab.edu/index.html) [19] database was used to analyze the relationship between the expression of *CENPL* mRNA and clinical characteristics including *TP53* mutation, age and sex. A *p* value of < 0.05 was considered statistically different.

*CENPL* gene expression and clinicopathological data regarding TCGA PAAD was from the UCSC Xena database (https://xenabrowser.net/) [20]. Univariate and multivariate Cox regression analyses were performed using survival package of R software (Version 3.6.3) to assess Hazard Ratio (HR) and *p*-values of prognostic risks for PAAD. The survminer package was used for visualization. The downloaded PAAD data included 181 patients' *CENPL* expression (FPKM) and clinicopathological data including sex, age, tumor size and survival. Of those, 82 were female and 99 were male, and 167 had tumor size data, with a median size of 3.5 cm, (Supplementary table 1).

### Mutation and levels of immune cell infiltration

The cBioPortal (https://www.cbioportal.org/) [21] database was used to analyze the mutations of *CENPL* in tumors. The relationship between *CENPL* mRNA and levels of immune cell infiltration was analyzed with the TIMER: Tumor Immune Estimation Resource (https://cistrome.shinyapps.io/timer/) [22] database. A *p* value of < 0.05 and a correlation coefficient value of ≥ 0.5 were considered statistically significant.

### Analysis of potential regulatory mechanisms

The miRWalk (http://mirwalk.umm.uni-heidelberg.de/) [23] database was used to predict potential microRNAs (miRNAs) that regulate *CENPL* mRNA. Cytoscape software (Version: 3.7.2) was used to screen and visualize top 9 potential miRNAs associated with CENPL expression. The Encyclopedia of RNA Interactomes (ENCORI, http://rna.sysu.edu.cn/encori/mirTarPathways.php) database was used to analyze the expression of miRNA candidates in cancers [24]. Pan-cancer Analysis Platform model was selected. A *p* value of < 0.05 were considered statistically different.

### Immunohistochemical Staining

The study was conducted in accordance with the principles of the Declaration of Helsinki [25]. The specimen collection procedure was approved by the Hospital Ethics Committee. The need for informed consent was waived by the Ethics Committee of Dongfang Hospital. The investigation was approved by the Ethics Committee of Dongfang Hospital.

Five-μm sections were obtained from paraffin-embedded PAAD tumor and non-tumor tissues (From Department of Pathology, Dongfang Hospital). All these sections were dewaxed in xylene and re-hydrated in alcohol followed by wet autoclave pretreatment in citrate buffer for antigen retrieval (10 min at 120℃, pH = 6.0) and then rinsed with phosphate buffer saline. Immunohistochemical staining for antibody to CENPL (Rabbit: bs13836R, Beijing Biosynthesis Biotechnology, China) was performed using the avidin–biotin- peroxidase complex method. The primary antibody (1:200 dilution for CENPL) was applied to the sections and allowed to react for 1 h at room temperature. The sections were then incubated with biotinylated anti-mouse/rabbit antibody for 30 min and avidin–biotin-peroxidase reagent for 20 min. After color development with diaminobenzidine, the sections were counterstained with hematoxylin.

## Results

### Levels of *CENPL* mRNA in ESCA, STAD, PAAD, COAD and READ

**Fig. 1:**
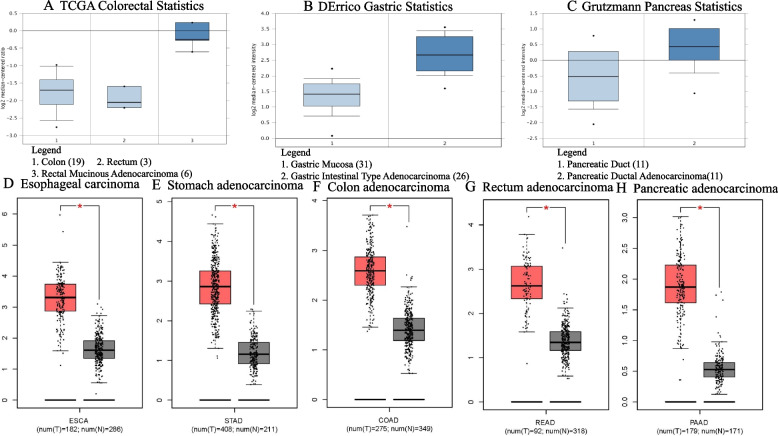
The level of *CENPL* mRNA in ESCA, STAD, COAD, READ and PAAD tumor and normal tissues. Higher levels of *CENPL* mRNA in gastric and colorectal cancers and PAAD tumor than normal tissues, *p* < 0.01, (**A**-**C**), (Oncomine) The level of *CENPL* mRNA in ESCA, STAD, COAD, READ and PAAD was higher than that in normal tissues, *p* < 0.01, (**D**-**H**), ( GEPIA).

Levels of *CENPL* mRNA in colorectal and gastric cancers and PAAD were higher than those in para-tumor tissues (*p* < 0.01 for all, Fig. 1A-C and Table 1). The range of fold change (FC) in COAD were 2.168–3.007 (Fig. 1A and Table 1). In STAD it was 2.194–2.502 (Fig. 1B and Table 1). FC was 2.039 in PAAD (Fig. 1C and Table 1). Using the defined threshold, no results were retrieved for esophageal carcinoma. Analysis of GEPIA database showed that the level of *CENPL* mRNA in 182 cases of ESCA was significantly higher than that in 286 cases of normal tissue (*p* < 0.01, Fig. 1D). *CENPL* mRNA was significantly overexpressed in 408 STAD cases than in 211 cases of normal tissues (*p* < 0.01, Fig. 1E). In 275 cases of COAD and 349 cases of normal tissues, *CENPL* mRNA was significantly higher in tumor (*p* < 0.01, Fig. 1F). Results of 92 cases of READ and 318 cases of normal tissues showed significantly increased levels of *CENPL* mRNA in tumors (*p* < 0.01, Fig. 1G). The expression of *CENPL* mRNA in 179 cases of PAAD was significantly higher than that in 171 cases of normal pancreatic tissues (*p* < 0.01, Fig. 1H).

**Table 1:** The FC of *CENPL* mRNA in gastrointestinal tumors (Oncomine)

**Cancer types**	**Datasets**	**Fold Change**	***p* Value**	***t* Test**
Colorectal adenocarcinoma	TCGA Colorectal Statistics			
	Rectal mucinous adenocarcinoma	3.007	3.22E-8	10.614
	Colon mucinous adenocarcinoma	2.406	1.19E-10	8.338
	Cecum adenocarcinoma	2.541	2.93E-11	8.798
	Rectosigmoid adenocarcinoma	2.893	3.15E-5	10.243
	Rectal adenocarcinoma	2.441	7.43E-14	11.296
	Colon adenocarcinoma	2.596	2.64E-14	12.595
	Sabates Bellver Colon et al.’s Statistics			
	Rectal adenoma	2.561	6.41E-8	8.996
	Colon adenoma	2.168	2.15E-9	7.254
Gastric adenocarcinoma	DErrico Gastric et al.’s Statistics			
	Gastric intestinal type adenocarcinoma	2.502	1.66E-12	9.078
	Gastric mixed adenocarcinoma	2.194	2.97E-4	6.656
PAAD	Grutzmann Pancreas et al.’s Statistics			
	Pancreatic ductal adenocarcinoma	2.039	0.002	3.255
Esophageal carcinoma	No	No	No	No

### Relationship between the level of *CENPL* mRNA and the prognosis of patients with ESCA, STAD, COAD, READ and PAAD

**Fig. 2:**
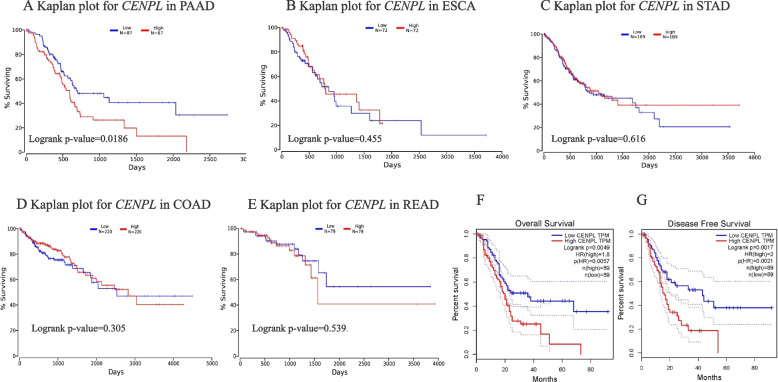
The prognostic value of *CENPL* mRNA in gastrointestinal tumors. PAAD patients with high levels of *CENPL* mRNA had shorter survival*p* < 0.05, (**A**), (OncoLnc). There was no significant correlationship between *CENPL* mRNA and the survival of patients with ESCA, STAD, COAD and READ, *p* > 0.05, (**B**-**E**), (OncoLnc). PAAD patients with higher levels of *CENPL* mRNA have shorter OS and DFS, *p* < 0.05, (**F**-**G**), (GEPIA)

In 174 patients with PAAD, those with higher levels of *CENPL* mRNA had shorter survival (*p* < 0.05, Fig. 2A). In 144 patients with ESCA, there was no significant difference in the survival between the high- and low-expression groups (*p* > 0.05, Fig. 2B). No significant difference was found in the survival of STAD patients between the high- and low-expression groups (*p* > 0.05, Fig. 2C). In 440 patients with COAD, no significant difference in the survival was observed between high- and low- expression groups (*p* > 0.05, Fig. 2D). There was no significant difference in the survival of READ patients between high- and low- expression groups (*p* > 0.05, Fig. 2E).

In addition, GEPIA was analyzed regarding the prognostic value of *CENPL* mRNA in patients with PAAD. The results showed that patients with higher levels of *CENPL* mRNA had shorter overall survival (OS) (*p* < 0.01, Fig. 2F). Similarly, patients with higher expression of *CENPL* mRNA had shorter disease-free survival (DFS) (*p* < 0.01, Fig. 2G).

**Fig. 3:**
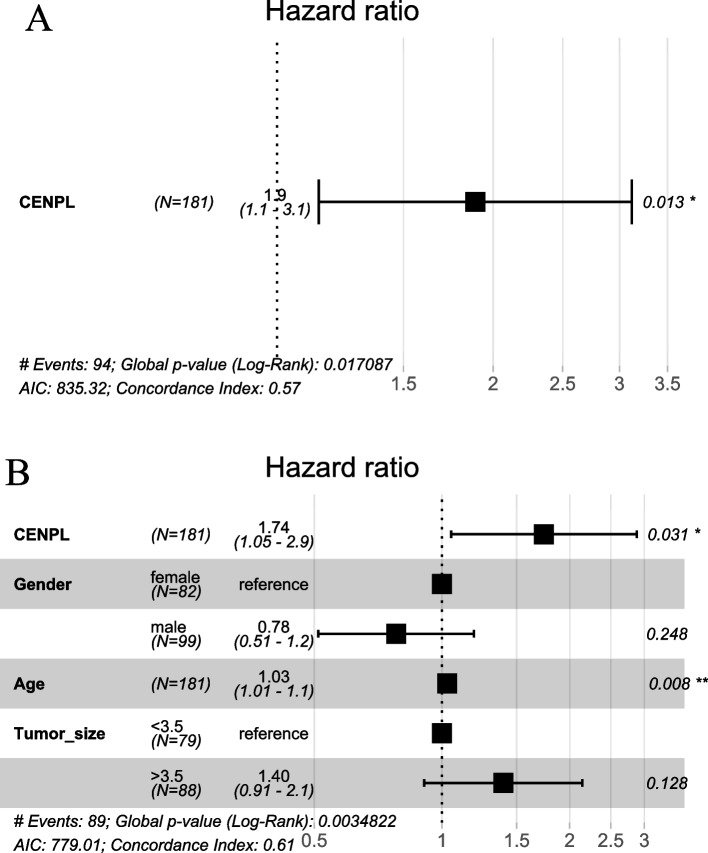
Univariate and multivariate Cox regression analyses of prognostic risk factors in PAAD patient. Univariate Cox regression analysis showed that *CENPL* mRNA was a risk factor for the prognosis of PAAD patients (HR = 1.9, *p* < 0.05) (**A**). Multivariate Cox regression analysis showed that *CENPL* and age were risk factors for patient prognosis (**B**), whereas sex and tumor size had no significant effects on the prognosis (**B**)

Univariate Cox regression analysis showed that *CENPL* mRNA was a risk factor for patient prognosis (HR = 1.9, CI = 1.1–3.1, *p* < 0.05) (Fig. 3A). Multivariate Cox regression analysis included *CENPL* mRNA, age, sex and tumor size (dichotomized with a cut-off value of 3.5 cm) and *CENPL* mRNA (HR = 1.74, CI = 1.05–2.9, *p* < 0.05) and age (HR = 1.03, CI = 1.01–1.1, *p* < 0.05) were independent risk factors for the prognosis of PAAD patients (Fig. 3B). Sex and tumor size were not independent risk factors forthe prognosis of PAAD patients (both *p* > 0.05) (Fig. 3B).

### *CENPL* mutation and the correlation between its expression and tumor infiltrating lymphocytes

**Fig. 4:**
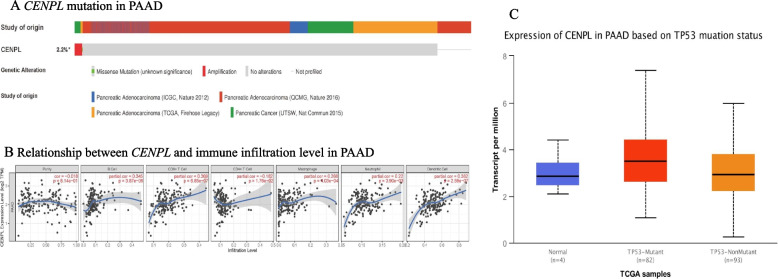
The mutations of *CENPL* and the relationship between the expression of *CENPL* mRNA and the abundance of tumor immune cell infiltration in PAAD. The mutation rate of *CENPL* in PAAD was 2% (17/850), (**A**), (cBioPortal). There was no significant correlation between the expression of *CENPL* mRNA and the abundance of immune cell infiltration in PAAD | Cor |< 0.5, (**B**), (TIMER). *CENPL* mRNA was significantly overexpressed in PAAD with *TP53* mutation, *p* < 0.01, (**C**), (UALCAN)

Gene mutation analysis showed that only 2% (17/850) of 850 patient from four independent datasets had mutations, and the main type of *CENPL* mutation was amplification (Fig. 4A). No significant correlations were observed between the expression of *CENPL* mRNA and PAAD tumor purity and levels of immune cell infiltration including B Cells, CD8 + T Cells, CD4 + T Cells, macrophages, neutrophils and dendritic cells (|Cor|< 0.5, Fig. 4B). However, it was found that the expression of *CENPL* mRNA was significantly associated with *TP53* mutations in PAAD. In 82 PAAD patients with *TP53* mutation, the level of *CENPL* mRNA was significantly higher than that in 93 patients without *TP53* mutation (*p* < 0.01, Fig. 4C).

### miRNAs potentially regulating *CENPL* in PAAD

**Fig. 5:**
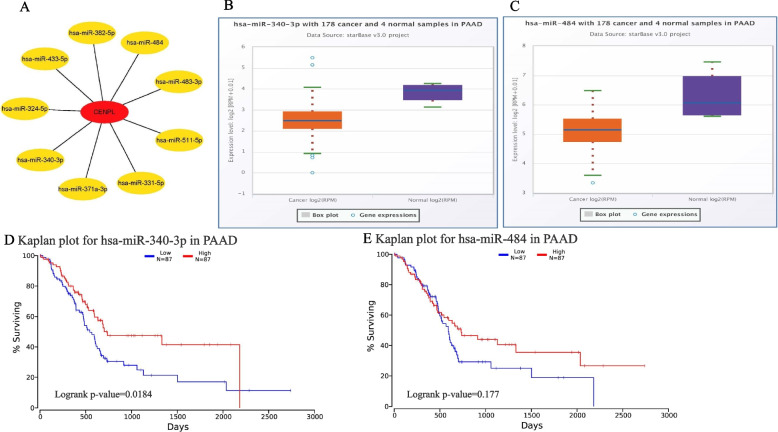
The miRNAs that potentially regulate the expression of *CENPL* mRNA in PAAD. The top 9 miRNAs with the potential to regulate *CENPL* mRNA expression in PAAD, (**A**), (miRWalk). miR-340-3p and miR-484 were significantly under-expressed in PAAD patients, (**B**-**C**), (ENCORI). PAAD patients with lower miR-340-3p expression have shorter survival (*p* < 0.05) (**D**), (OncoLnc). There was no significantly correlation between miR-484 expression and the survival of PAAD patients (*p* > 0.05, OncoLnc) (E)

1086 miRNAs (supplement table 2) that potentially regulate *CENPL* mRNA were identified using the miRWalk database. Cytoscape (Version: 3.7.2) screened the top 9 potential miRNAs with the strongest association with *CENPL* mRNA: hsa-miR-371a-3p, hsa-miR-382-5p, hsa-miR-340-3p, hsa- miR-331-5p, hsa-miR-324-5p, hsa-miR-433-5p, hsa-miR-483-3p, hsa-miR-484 and hsa-miR-511-5p (Fig. 5A). Further analysis showed significant low expression of hsa-miR-340-3p in PAAD (FC = 0.44, *p* < 0.01 and FDR < 0.05, Fig. 5B). hsa-miR-484 was also significantly under-expressed (FC = 0.41, *p* < 0.01 and FDR < 0.05, Fig. 5C). Moreover, it was found that PAAD patients with lower expression of hsa-miR-340-3p had shorter survival (*p* < 0.05, Fig. 5D). The expression of hsa-miR-484 was not significantly correlated with the survival of PAAD patients (*p* = 0.177, Fig. 5E).

### The expression of CENPL protein in PAAD and normal tissues

**Fig. 6:**
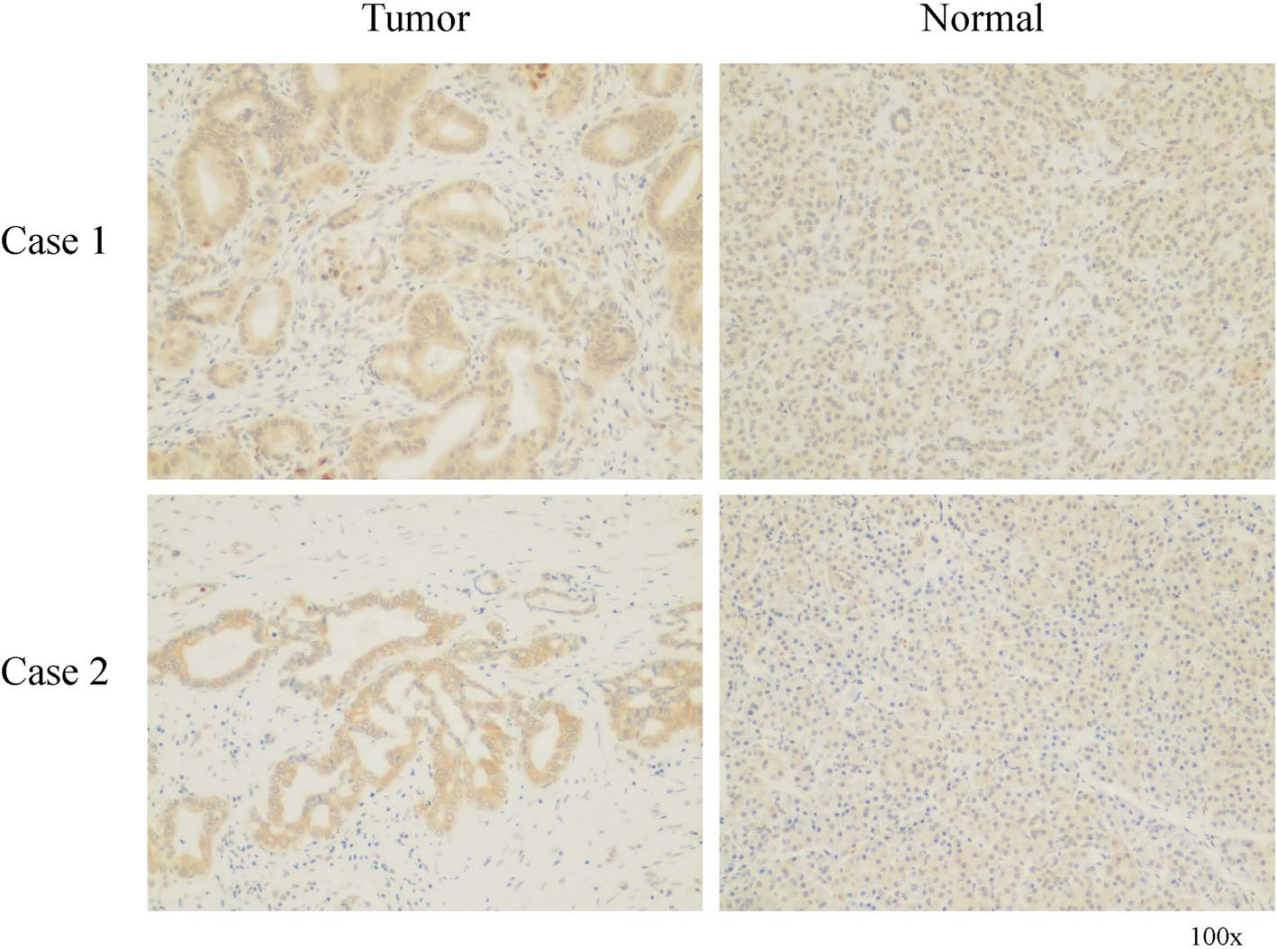
The expression of CENPL protein in PAAD tumor and normal tissues. Immunohistochemical staining showed that the expression of CENPL protein in tumor was higher than in normal tissues

Immunohistochemical staining of clinical specimens showed that the expression of CENPL protein in PAAD was significantly higher than that in adjacent normal tissues. The expression of CENPL in tumor tissues (42%, 11/26) was higher than that in non-cancer tissues (Fig. 6). CENPL protein was mainly located in cytoplasm and membrane (Fig. 6).

## Discussion

In this study, the expression of *CENPL* mRNA in gastrointestinal tumors was analyzed using multiple public databases. *CENPL* mRNA was overexpressed in ESCA, STAD, PAAD, COAD and READ tumors compared with adjacent normal tissues. Then, the prognostic value of *CENPL* in patients with gastrointestinal tumors was assessed. The level of *CENPL* mRNA significantly predicted the prognosis of PAAD patients. Univariate and multivariate Cox regression analyses showed that *CENPL* mRNA was an independent risk factor for prognosis, suggesting that *CENPL* may play a critical role in PAAD and is worthy of further study. The mutation rate of *CENPL* was low and the relationship was not significant between *CENPL* expression and the abundance of immune cell infiltration in PAAD. However, it was found that the level of *CENPL* mRNA was significantly correlated with *TP53* mutation in PAAD. Immunotherapy plays an important role in the treatment of cancer [26]. Therefore, we analyzed the expression level of *CENPL* mRNA in skin cutaneous melanoma (SKCM), lung adenocarcinoma (LUAD), lung squamous cell carcinoma (LUSC), kidney renal clear cell carcinoma (KIRC), breast invasive carcinoma (BRCA), and bladder urothelial carcinoma (BLCA) by GEPIA [17], for which immunotherapy have been approved. The results showed that the expression of *CENPL* mRNA was significantly increased in SKCM, LUAD, LUSC and BRCA, and no significant overexpression in KIRC and BLCA (Supplement Fig. 1). This also suggests that *CENPL* may exert effects in a variety of tumors. miR-340-3p was not only significantly under-expressed in PAAD, but also associated with worse prognosis in these patients, suggestive of a potential role in regulation of *CENPL* expression in PAAD. Finally, it was verified that CENPL protein was overexpressed in many clinical specimens from PAAD patients.

PAAD is the seventh leading cause of cancer-related deaths worldwide. Notably, the incidence of PAAD is growing, and the patient survival has barely improved [6, 27]. The pathogenesis, early diagnosis and precise treatment remain challenging. Studies have revealed that *TP53* mutation occurred in approximately 70% of patients with PAAD and was one of the major factors involved in development and progression of PAAD [28–31]. In this study, *CENPL* was rarely mutated in PAAD. However, the level of *CENPL* mRNA was higher in patients with *TP53* mutation. These results suggest that *CENPL* may have a robust association with *TP53* mutations in PAAD patients. CENPL is one of the proteins that constitute kinetosome, and overexpressed CENPL may affect the function of kinetochore. Previous studies have shown that kinetosome dysfunction might be a critical cause of chromosome instability and tumorigenesis [32–34]. Therefore, we hypothesize that overexpression of CENPL impairs the function of kinetosome and cause chromosomal instability, thereby causing or accelerating TP53 mutation in pancreatic cancer. which is worthy of further exploration.

miRNA is a single-stranded non-coding RNA molecule with a length ranging from 19 to 25 nucleotides. It is encoded by the endogenous gene. miRNA is involved in biological functions of cells by regulating its target genes [35]. miRNAs widely participate in signal activation, and cell proliferation, differentiation and death [35]. miR-484 is a key factor in cancer and non-cancerous diseases, and its targets in cancer include VEGFB, VEGFR2, MAP2, MMP14, HNF1A, TUSC5, and KLF12 [36]. miR-484 was found to precipitate tumorigenesis and metastasis in liver, prostate and lung cancers [36]. Nan et al. reported that in pancreatic ductal adenocarcinoma, the inhibitory effect of miR-484 on YAP was attenuated and predicted adverse patient outcomes [37]. In this study, we found that *CENPL* overexpression in PAAD might be the consequence of miR-484 suppression.

Previous studies have shown that miR-340 acted as an oncogenic or tumor suppressor by targeting genes related to proliferation, apoptosis, and metastasis. It showed significance in diagnosis, treatment, chemotherapy resistance, and prognosis [38]. Two studies have found that miR-340-3p might be closely related to the development of laryngeal squamous cell carcinoma and breast cancer [39, 40]. A recent study showed that miR-340-3p regulated by lncRNA HOXA10-AS could down-regulate HTR1D and inhibit the malignant biological behavior of pancreatic cancer through PI3K-Akt signaling pathway [41]. In this study, it was found to have a potential role in PAAD by targeting *CENPL*. In other words, the overexpression of *CENPL* mRNA in PAAD may be caused by insufficient miR-340-3p. Therefore, it may be a critical molecule in the pathway that regulates the expression of *CENPL*. Furthermore, by using the GEPIA [17] and DAVID [42, 43] databases, we found the top 200 genes that were significantly co-expressed with *CENPL* in PAAD, mainly enriched in endocytosis (Supplement table 3). Endocytosis was revealed to be a pivotal pathway for regulation of metastasis [44]. Therefore, the overexpression of CENPL in PAAD may be involved in this process leading to disease deterioration.

There are some limitations in this study. Laboratory studies of the function of CENPL in PAAD cells is lacking, and the regulatory effects of miR-340-3p on *CENPL* mRNA expression should be verified through experiments. Furthermore, the number of PAAD patients involved in immunohistochemistry was small.

## Conclusion

In summary, the current study suggests that *CENPL* may play an important pathologic role in PAAD, and *CENPL* mRNA is a prognostic predictor for PAAD patients.

## Supplementary information

**Additional file 1.**

**Additional file 2.**

**Additional file 3.**

**Additional file 4.**

## Data Availability

The datasets generated and/or analysed during the current study are available in the GEPIA, OncoLnc, UALCAN, cBioPortal, UCSC Xena, TIMER, miRWalk and ENCORI repository. [GEPIA: (http://gepia.cancer-pku.cn/detail.php?gene=&clicktag=boxplot); OncoLnc: (http://www.oncolnc.org/kaplan/?lower=50&upper=50&cancer=PAAD&gene_id=91687&raw=CENPL&species=mRNA), (http://www.oncolnc.org/kaplan/?lower=50&upper=50&cancer=ESCA&gene_id=91687&raw=CENPL&species=mRNA), (http://www.oncolnc.org/kaplan/?lower=50&upper=50&cancer=STAD&gene_id=91687&raw=CENPL&species=mRNA), (http://www.oncolnc.org/kaplan/?lower=50&upper=50&cancer=COAD&gene_id=91687&raw=CENPL&species=mRNA), (http://www.oncolnc.org/kaplan/?lower=50&upper=50&cancer=READ&gene_id=91687&raw=CENPL&species=mRNA), (http://www.oncolnc.org/kaplan/?lower=50&upper=50&cancer=PAAD&gene_id=MIMAT0000750&raw=hsa-miR-340-3p&species=miRNA) (http://www.oncolnc.org/kaplan/?lower=50&upper=50&cancer=PAAD&gene_id=MIMAT0002174&raw=hsa-miR-484&species=miRNA); UALCAN: (http://ualcan.path.uab.edu/cgi-bin/TCGAExResultNew2.pl?genenam=CENPL&ctype=PAAD); cBioPortal: (https://www.cbioportal.org/results/oncoprint?cancer_study_list=paad_icgc%2Cpaad_qcmg_uq_2016%2Cpaad_utsw_2015%2Cpaad_tcga&Z_SCORE_THRESHOLD=2.0&RPPA_SCORE_THRESHOLD=2.0&profileFilter=mutations%2Cgistic&case_set_id=all&gene_list=CENPL&geneset_list=%20&tab_index=tab_visualize&Action=Submit); UCSC Xena: (https://xenabrowser.net/datapages/?cohort=GDC%20TCGA%20Pancreatic%20Cancer%20(PAAD)&removeHub=https%3A%2F%2Fxena.treehouse.gi.ucsc.edu%3A443); TIMER: (https://cistrome.shinyapps.io/timer/); miRWalk: (http://mirwalk.umm.uni-heidelberg.de/human/gene/91687/) and ENCORI: (https://rna.sysu.edu.cn/encori/panMirDiffExp.php)].

## References

[1] Bray F, Ferlay J, Soerjomataram I, Siegel RL, Torre LA, Jemal A (2018). Global cancer statistics 2018: GLOBOCAN estimates of incidence and mortality worldwide for 36 cancers in 185 countries. CA Cancer J Clin.

[2] Arnold M, Abnet CC, Neale RE, Vignat J, Giovannucci EL, McGlynn KA (2020). Global Burden of 5 Major Types of Gastrointestinal Cancer. Gastroenterology.

[3] Thrift AP, El-Serag HB (2020). Burden of Gastric Cancer. Clin Gastroenterol Hepatol.

[4] Siegel RL, Miller KD, Goding Sauer A, Fedewa SA, Butterly LF, Anderson JC (2020). Colorectal cancer statistics, 2020. CA Cancer J Clin.

[5] Wong MCS, Huang J, Lok V, Wang J, Fung F, Ding H (2021). Differences in Incidence and Mortality Trends of Colorectal Cancer Worldwide Based on Sex, Age, and Anatomic Location. Clin Gastroenterol Hepatol.

[6] Huang J, Lok V, Ngai CH, Zhang L, Yuan J, Lao XQ (2021). Worldwide Burden of, Risk Factors for, and Trends in Pancreatic Cancer. Gastroenterology.

[7] Abdul-Latif M, Townsend K, Dearman C, Shiu KK, Khan K (2020). Immunotherapy in gastrointestinal cancer: The current scenario and future perspectives. Cancer Treat Rev.

[8] Nakamura Y, Taniguchi H, Ikeda M, Bando H, Kato K, Morizane C (2020). Clinical utility of circulating tumor DNA sequencing in advanced gastrointestinal cancer: SCRUM-Japan GI-SCREEN and GOZILA studies. Nat Med.

[9] Zhang X, Wu Z, Peng Y, Li D, Jiang Y, Pan F (2021). Correlationship between Ki67, VEGF, and p53 and Hepatocellular Carcinoma Recurrence in Liver Transplant Patients. Biomed Res Int.

[10] Hara M, Fukagawa T (2020). Dynamics of kinetochore structure and its regulations during mitotic progression. Cell Mol Life Sci.

[11] Kursel LE, Malik HS (2016). Centromeres. Curr Biol.

[12] McKinley KL, Sekulic N, Guo LY, Tsinman T, Black BE, Cheeseman IM (2015). The CENP-L-N Complex Forms a Critical Node in an Integrated Meshwork of Interactions at the Centromere-Kinetochore Interface. Mol Cell.

[13] McHedlishvili N, Wieser S, Holtackers R, Mouysset J, Belwal M, Amaro AC (2012). Kinetochores accelerate centrosome separation to ensure faithful chromosome segregation. J Cell Sci.

[14] Yin J, Lin C, Jiang M, Tang X, Xie D, Chen J (2021). CENPL, ISG20L2, LSM4, MRPL3 are four novel hub genes and may serve as diagnostic and prognostic markers in breast cancer. Sci Rep.

[15] Cui Z, Xiao L, Chen F, Wang J, Lin H, Li D (2021). High mRNA Expression of CENPL and Its Significance in Prognosis of Hepatocellular Carcinoma Patients. Dis Markers.

[16] Rhodes DR, Yu J, Shanker K, Deshpande N, Varambally R, Ghosh D (2004). ONCOMINE: a cancer microarray database and integrated data-mining platform. Neoplasia.

[17] Tang Z, Li C, Kang B, Gao G, Li C, Zhang Z (2017). GEPIA: a web server for cancer and normal gene expression profiling and interactive analyses. Nucleic Acids Res.

[18] OncoLnc JA (2016). linking TCGA survival data to mRNAs, miRNAs, and lncRNAs. PeerJ Computer Science.

[19] Chandrashekar DS, Bashel B, Balasubramanya SAH, Creighton CJ, Ponce-Rodriguez I, Chakravarthi B (2017). UALCAN: A Portal for Facilitating Tumor Subgroup Gene Expression and Survival Analyses. Neoplasia.

[20] Goldman MJ, Craft B, Hastie M, Repecka K, McDade F, Kamath A (2020). Visualizing and interpreting cancer genomics data via the Xena platform. Nat Biotechnol.

[21] Cerami E, Gao J, Dogrusoz U, Gross BE, Sumer SO, Aksoy BA (2012). The cBio cancer genomics portal: an open platform for exploring multidimensional cancer genomics data. Cancer Discov.

[22] Li T, Fan J, Wang B, Traugh N, Chen Q, Liu JS (2017). TIMER: A Web Server for Comprehensive Analysis of Tumor-Infiltrating Immune Cells. Cancer Res.

[23] Sticht C, De La Torre C, Parveen A, Gretz N (2018). miRWalk: An online resource for prediction of microRNA binding sites. PLoS ONE.

[24] Li JH, Liu S, Zhou H, Qu LH, Yang JH. starBase v2.0: decoding miRNA-ceRNA, miRNA-ncRNA and protein-RNA interaction networks from large-scale CLIP-Seq data. Nucleic Acids Res. 2014;42(Database issue):D92–7. 10.1093/nar/gkt1248 PMC3964941 24297251

[25] World Medical A (2013). World Medical Association Declaration of Helsinki: ethical principles for medical research involving human subjects. JAMA.

[26] Yang L, Ning Q, Tang SS (2022). Recent Advances and Next Breakthrough in Immunotherapy for Cancer Treatment. J Immunol Res.

[27] Khalaf N, El-Serag HB, Abrams HR, Thrift AP (2021). Burden of Pancreatic Cancer: From Epidemiology to Practice. Clin Gastroenterol Hepatol.

[28] Weissmueller S, Manchado E, Saborowski M, Morris JPt, Wagenblast E, Davis CA, et al. Mutant p53 drives pancreatic cancer metastasis through cell-autonomous PDGF receptor beta signaling. Cell. 2014;157(2):382–94. 10.1016/j.cell.2014.01.066 PMC4001090 24725405

[29] Morton JP, Timpson P, Karim SA, Ridgway RA, Athineos D, Doyle B (2010). Mutant p53 drives metastasis and overcomes growth arrest/senescence in pancreatic cancer. Proc Natl Acad Sci U S A.

[30] Hashimoto S, Furukawa S, Hashimoto A, Tsutaho A, Fukao A, Sakamura Y (2019). ARF6 and AMAP1 are major targets of KRAS and TP53 mutations to promote invasion, PD-L1 dynamics, and immune evasion of pancreatic cancer. Proc Natl Acad Sci U S A.

[31] van der Sijde F, Azmani Z, Besselink MG, Bonsing BA, de Groot JWB, Groot Koerkamp B (2021). Circulating TP53 mutations are associated with early tumor progression and poor survival in pancreatic cancer patients treated with FOLFIRINOX. Ther Adv Med Oncol.

[32] Yuen KW, Montpetit B, Hieter P (2005). The kinetochore and cancer: what's the connection?. Curr Opin Cell Biol.

[33] Jo M, Kusano Y, Hirota T (2021). Unraveling pathologies underlying chromosomal instability in cancers. Cancer Sci.

[34] Dong Q, Yang J, Gao J, Li F (2021). Recent insights into mechanisms preventing ectopic centromere formation. Open Biol.

[35] Hammond SM (2015). An overview of microRNAs. Adv Drug Deliv Rev.

[36] Jia YZ, Liu J, Wang GQ, Song ZF (2022). miR-484: A Potential Biomarker in Health and Disease. Front Oncol.

[37] Li N, Yang G, Luo L, Ling L, Wang X, Shi L (2020). lncRNA THAP9-AS1 Promotes Pancreatic Ductal Adenocarcinoma Growth and Leads to a Poor Clinical Outcome via Sponging miR-484 and Interacting with YAP. Clin Cancer Res.

[38] Huang Z, Xu Y, Wan M, Zeng X, Wu J (2021). miR-340: A multifunctional role in human malignant diseases. Int J Biol Sci.

[39] Yan L, Yang S, Yue CX, Wei XY, Peng W, Dong ZY (2020). Long noncoding RNA H19 acts as a miR-340-3p sponge to promote epithelial-mesenchymal transition by regulating YWHAZ expression in paclitaxel-resistant breast cancer cells. Environ Toxicol.

[40] Shuang Y, Liu J, Niu J, Guo W, Li C (2021). A novel circular RNA circPPFIA1 promotes laryngeal squamous cell carcinoma progression through sponging miR-340-3p and regulating ELK1 expression. Bioengineered.

[41] Wu W, Li Q, Zhu Z, Li C, Lu P, Zhou X (2022). HTR1D functions as a key target of HOXA10-AS/miR-340-3p axis to promote the malignant outcome of pancreatic cancer via PI3K-AKT signaling pathway. Int J Biol Sci.

[42] da Huang W, Sherman BT, Lempicki RA (2009). Systematic and integrative analysis of large gene lists using DAVID bioinformatics resources. Nat Protoc.

[43] Sherman BT, Hao M, Qiu J, Jiao X, Baseler MW, Lane HC, et al. DAVID: a web server for functional enrichment analysis and functional annotation of gene lists (2021 update). Nucleic Acids Res. 2022. 10.1093/nar/gkac194 PMC9252805 35325185

[44] Khan I, Steeg PS (2021). Endocytosis: a pivotal pathway for regulating metastasis. Br J Cancer.

