# Correction: Overexpression of *CENPL* mRNA potentially regulated by miR-340-3p predicts the prognosis of pancreatic cancer patients

**DOI:** 10.1186/s12885-023-10504-2

**Published:** 2023-01-05

**Authors:** Zhongyuan Cui, Ling Du, Jielong Wang, Zhongzhuan Li, Jiehong Xu, Shiyu Ou, Dongliang Li, Shasha Li, Hanfang Hu, Gang Chen, Zhixian Wu

**Affiliations:** 1 Department of Hepatobiliary Disease, 900th Hospital of the Joint Logistics Support Force (Dongfang Hospital), Xiamen University, Fuzhou, 350025 Fujian China; 2 Department of Gastroenterology, the Fourth Afliated Hospital (Liuzhou Workers’ Hospital), Guangxi Medical University, Liuzhou, 545000 Guangxi China; 3 Department of Hepatobiliary Disease, 900th Hospital of the Joint Logistics Support Force, Fujian Medical University, Fuzhou, 350025 Fujian China

**Correction: BMC Cancer 22, 1354 (2022)**

**https://doi.org/10.1186/s12885-022-10450-5**

Following publication of the original article [1], the authors reported an error in the affiliation of Zhixian Wu. Zhixian Wu is affiliated to institution 1 and 3. The affiliations in this correction article have been updated and the original article [1] has been corrected.
