# Supplementary material for: Overexpression of CENPL mRNA potentially regulated by miR-340-3p predicts the prognosis of pancreatic cancer patients
Source: BMC Cancer. 2022 Dec 26;22:1354. doi: 10.1186/s12885-022-10450-5 (PMC9793567; doi:10.1186/s12885-022-10450-5)
Supplement: Supplementary file 1 — Additional file 1. [file 12885_2022_10450_MOESM1_ESM.pptx]

## Slide 1
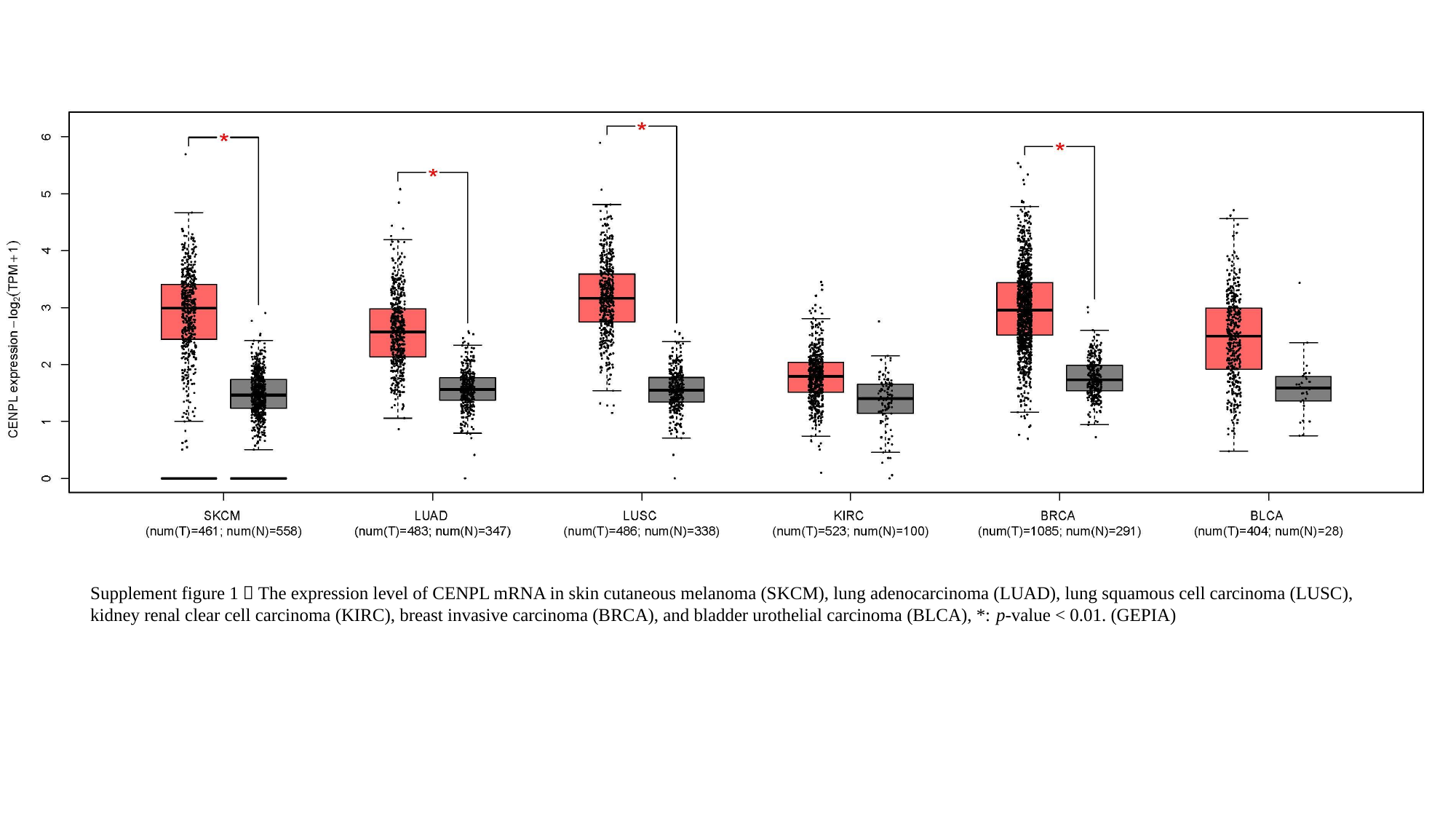

Supplement figure 1：The expression level of CENPL mRNA in skin cutaneous melanoma (SKCM), lung adenocarcinoma (LUAD), lung squamous cell carcinoma (LUSC), kidney renal clear cell carcinoma (KIRC), breast invasive carcinoma (BRCA), and bladder urothelial carcinoma (BLCA), *: p-value < 0.01. (GEPIA)
